# Supplementary material for: From sequence to enzyme mechanism using multi-label machine learning
Source: BMC Bioinformatics. 2014 May 19;15:150. doi: 10.1186/1471-2105-15-150 (PMC4229970; doi:10.1186/1471-2105-15-150)
Supplement: Additional file 2 — Java code of ml2db. Additional file ml2db_code.tar.gz contains the Java source code to run the multi-label machine learning experiments and save the results to database. The code’s Javadoc is included. [file 1471-2105-15-150-S2.zip › additional file 2/ml2db/utils/doc/uk/ac/standrews/utils/main/database/DbCreator.html]

DbCreator


JavaScript is disabled on your browser.


- Overview
- Package
- Class
- Use
- Tree
- Deprecated
- Index
- Help

- Prev Class
- Next Class

- Frames
- No Frames

- All Classes

- Summary:
- Nested |
- Field |
- Constr |
- Method

- Detail:
- Field |
- Constr |
- Method


uk.ac.standrews.utils.main.database

## Class DbCreator

- java.lang.Object
- - uk.ac.standrews.utils.main.database.DbManaged
  - - uk.ac.standrews.utils.main.database.DbCreator

- ---

    

  ```
  public class DbCreator
  extends DbManaged
  ```

  Class

  Version:
  :   20 Feb 2009

  Author:
  :   Luna De Ferrari luna.deferrari-at-ed.ac.uk

- - ### Constructor Summary

    Constructors

    Constructor and Description

    `DbCreator(DbManager dbManager)`
  - ### Method Summary

    Methods

    | Modifier and Type | Method and Description |
    | --- | --- |
    | `void` | `createTables()` |
    | `boolean` | `dropTable(java.lang.String tableName)` Drops the database table. |
    | `java.lang.String` | `getSqlToDropTable(java.lang.String tableName)` Get sql command to drop table |

    - ### Methods inherited from class uk.ac.standrews.utils.main.database.DbManaged

      `getDbConnection, getDbManager, getDbReader`
    - ### Methods inherited from class java.lang.Object

      `equals, getClass, hashCode, notify, notifyAll, toString, wait, wait, wait`

- - ### Constructor Detail


    - #### DbCreator

      ```
      public DbCreator(DbManager dbManager)
      ```
  - ### Method Detail


    - #### createTables

      ```
      public void createTables()
      ```


    - #### dropTable

      ```
      public boolean dropTable(java.lang.String tableName)
      ```

      Drops the database table. Beware! risk of data loss. Better to rename the
      old table and relaunch than to drop the table.

      Returns:
      :   true if the table does not exist in the database anymore


    - #### getSqlToDropTable

      ```
      public java.lang.String getSqlToDropTable(java.lang.String tableName)
      ```

      Get sql command to drop table

      Returns:
      :   the drop table SQL command


- Overview
- Package
- Class
- Use
- Tree
- Deprecated
- Index
- Help

- Prev Class
- Next Class

- Frames
- No Frames

- All Classes

- Summary:
- Nested |
- Field |
- Constr |
- Method

- Detail:
- Field |
- Constr |
- Method
